# Comments on the nonpharmaceutical interventions in New York City and Chicago during the 1918 flu pandemic

**DOI:** 10.1186/1479-5876-5-65

**Published:** 2007-12-11

**Authors:** John M Barry

**Affiliations:** 1 Center for Bioenvironmental Research, Tulane University, New Orleans, LA, USA

## Abstract

This commentary was originally published in CIDRAP News and it is here reported almost verbatim to allow divulgation through open access. The Editorial summarizes John Barry's concerns about the value of accurate historical reporting and its implications in public policy determination. This short abstract was written by the Editor-in-Chief of the Journal of Translational Medicine to introduce the Editorial.

## Editorial

### Remarks from CIDRAP Director Michael T. Osterholm, PhD, MPH

John M. Barry is one of our nation's most respected historians, and his chronicle of the 1918 pandemic, *The Great Influenza: The Story of the Deadliest Pandemic in History*, is regarded by many influenza and public health experts as the definitive historical review of that event [1,2]. To paraphrase the old E.F. Hutton commercial, "When Barry speaks, most of us concerned about our preparedness for the next pandemic listen." You will find below a commentary from Barry dealing with the important question of the use of nonpharmaceutical measures during the 1918 pandemic.

As any reader of this Web site knows, CIDRAP has made pandemic influenza preparedness one of its highest priorities as an infectious disease research and policy organization. I believe the next influenza pandemic, if even moderate in nature, will be one of the most catastrophic public health events in history. I come to this conclusion because of the size of the world's population today (approximately 6.5 billion, compared with 1.2 billion in 1918), the likelihood that there will be a lack of stockpiled and effective pandemic vaccine at outset of the pandemic, and the existence of the global just-in-time economy, which means we will soon exhaust many critical products and services, like drugs and vaccines, other medical supplies, and even food, in the early days of the pandemic.

Frankly, our one real hope is that all the other public health tools we have employed in past infectious disease epidemics will make a difference. These tools have largely tried to change individual and community-based behavior to avoid exposure to the infectious agent until after the epidemic has run its course. These are often referred to as nonpharmaceutical interventions (NPIs) and include familiar approaches such as isolation, quarantine, and social distancing. While all of us might believe that these measures will work, until recently very little evidence has been available concerning their efficacy in reducing either morbidity or mortality in an influenza pandemic. This is due in part to the infrequency of such pandemics (three in the last century) and an absence of systematic studies during those pandemics of our collective public health actions and their impact.

This past August, however, Howard Markel of the University of Michigan and colleagues published a study of the public health actions taken in 43 cities during the 1918–19 pandemic and the associated morbidity and mortality in those cites [3]. They concluded that when NPI strategies were employed, they made a difference. This was a very important conclusion, as the results of the Markel study now serve as the core support for the recent Centers for Disease Control and Prevention recommendations for the use of community mitigation strategies (ie, NPIs) during a pandemic [4].

Since publication of his book in 2004, Barry has been involved in the preparedness effort and continued to do research on NPIs. Most of this work has expanded on findings in the book, and some of it has caused him to revise views expressed there. Since the Markel publication in *JAMA*, Barry has raised serious challenges to the data used by Markel's group to justify their conclusions about the public health actions they reported to have been taken in 2 of the 43 cities (New York City and Chicago). These are the only two cities among the 43 for which Barry did such follow-up research. Barry wrote what I believe to be a convincing and well-supported letter to *JAMA *with his concerns. Last week his letter [5] and Markel's response were published in *JAMA *[6].

In the letters, Barry debated with Markel and colleagues whether New York City authorities ever used isolation and quarantine to combat the influenza pandemic in the fall of 1918.

Barry acknowledged that City Health Commissioner Royal Copeland told reporters in September 1918 that he would use isolation and quarantine. However, he cited a pair of articles published in an October 1918 issue of the *New York Medical Journal *by Copeland and his assistant as evidence that these measures were never used. Both articles reported on the epidemic and the city's response, but neither mentioned isolation, quarantine, or other major NPIs. Further, the city health department's annual report mentioned no NPIs used in the flu epidemic, according to Barry.

In response, Markel and his colleagues cited news reports from the *New York Times *and *JAMA *as evidence that the city did use isolation and quarantine. A story in the *Times *for Sep 19, 1918, said three New Yorkers had been quarantined the day before, and in a Nov 17 story, Copeland was quoted as saying the city had quarantined arriving ship passengers. In addition, a story in the Sep 28 *JAMA *said flu patients were "quarantined," according to Markel et al. They also noted that Barry cited the *JAMA *story in his own book as evidence of a New York City quarantine.

I believe Markel and colleagues did not address the important challenges that Barry presented. In my view, his information raises serious challenges to the scientific integrity of what Markel and colleagues have reported for two cites included in their study, which in turn raises important questions about the overall results of their study. This concern does not disprove that NPIs altered the course of the pandemic. But we in public health will face overwhelming challenges with risk communication and credibility during the next pandemic. While we will surely recommend the use of NPIs at that time, we have an obligation to society to tell exactly what we know and explain the science that supports our conclusions. How will we ever be able to dismiss and even condemn the crazy things that some will try to do during a pandemic if we don't base recommendations on the strength of our science? We must hold ourselves to that standard now and in the future. I believe John Barry makes a clear and compelling case below that Markel has not met that standard. *We *must.

### Commentary by John M. Barry

Policy makers are today considering implementing nonpharmaceutical interventions in the face of a pandemic on the basis of analyses of their historic use in 1918. I support many (though not all) of the proposed interventions, but I do not support analysis based on weak data – especially when those data are flatly contradicted by better information. Yet that is what could happen in this case. And although my letter to *JAMA *[5] was limited to New York City, the mistaken data in the article by Markel et al [3]seem to extend at least to Chicago as well.

It is not difficult to establish the facts, but Markel in his reply to my letter to *JAMA *[6] manages to obscure them. He cites two sources in addition to the *New York Times *to support his claim that New York City imposed a quarantine. These other sources are a 1918 *JAMA *article and my own book. Yet in actuality these other citations, as well as the *New York Times *itself, all circle back to a single person – Royal Copeland, head of the city's health department – and what he told the *Times*. Everything the newspaper reported about quarantine, including Markel's claim that the "next day, 3 New Yorkers were quarantined," came from a direct quote by Copeland; the newspaper did no independent reporting whatsoever, nor did it quote any other health department or city official, or anyone else, in these stories on quarantine. (More about Copeland later.) The 1918 *JAMA *citation is not a peer-reviewed article; it is a news item based on Copeland's published comments.

Markel also cited my book. In my book research, I read the same *New York Times *and *JAMA *articles that he did, and I initially believed them, as he still does. Hence my book incorrectly stated that New York imposed a quarantine. However, I gave the public health response in New York City only the most cursory consideration in the book. All my focus, and all my digging into primary sources and archival material, lay elsewhere. After my book was published, and as I got involved with the pandemic preparedness effort, the fact that New York City had a relatively benign experience continued to intrigue me. Since New York City did not close schools, I became increasingly curious if its quarantine could have accounted for its experience. So I investigated. New data convinced me that there was no quarantine, and I reversed my position. The footnotes and bibliography to his original article suggest that Markel never learned of these data, some of which are detailed below.

Here are the undisputed facts:

### Records don't mention quarantine

Markel makes much of the city Board of Health's vote to make influenza a reportable disease on Sep 17. The board did make this decision, but this does not support his argument. Making a disease reportable does absolutely nothing in itself to control that disease. Neither the minutes of the Sep 17 board meeting nor the minutes of other board meetings throughout the pandemic mention quarantine, although they do include discussion of less severe NPIs that were employed. This is a strange omission if quarantine was actually used. Nor do any other department records contain any evidence that the city imposed quarantine. Had there been a quarantine, one would have expected details about how many inspectors were involved, the number of homes visited, the period of quarantine, placards put up, etc. Health departments in cities that did impose quarantine recorded all these data. Yet no such data exist for New York City. Quarantine was not mentioned in the weekly, monthly, or annual reports of the heath department, even though these reports were so detailed that, as I pointed out in my original letter, they recounted how many laboratory flasks were washed.

The two articles in the *New York Medical Journal *– they were reprints of speeches, as Markel says – are primary sources; they are contemporaneous reports to physicians gathered to discuss the outbreak by Copeland and his deputy Louis Harris, head of the department's Bureau of Preventable Disease. Harris gave the presentation reprinted under the title "Epidemiology and Administrative Control of Influenza," which is one of the items missing from Markel's bibliography. His unfamiliarity with this source is a serious lapse, since the report explicitly addresses what we now call NPIs in New York City and was authored by the person in charge of those interventions, including all department quarantines. Both men recite a long list of actions taken, beginning with some attempt during the summer to monitor a few individual case-patients coming off ships.

For the sake of argument, let's say Markel is correct and this summer effort was more robust than I think. That still has nothing to do with what happened during the outbreak itself. It's one thing to surveil three or four people coming off a ship, especially since no effort was made to monitor any asymptomatic individuals, a failure which rather undermines any attempt to control disease such as influenza. It's another to wait for a case to be reported (and hope cases are in fact being reported) and then try to isolate dozens, then quickly hundreds, then thousands of influenza cases. No wonder Copeland debunked the idea, as he did in the quotation used in my letter to *JAMA*. Markel accuses me of taking this quote out of context. Rather than argue with him, I will happily fax the articles to anyone who requests them (print quality of the articles prevents including a link here) so readers can judge for themselves.

According to the *New York Medical Journal *articles, as the pandemic began and after making the disease reportable, the health department launched a series of initiatives. Harris lists them, and they include but are not limited to public education efforts (which proved disappointing to both Copeland and Harris), enforcement of antispitting ordinances, an attempt to stagger business closings to limit rush-hour crowds, closing of selected unsanitary theaters, and the like. These articles also justified the decisions to keep schools open and not to ban public gatherings, as most other cities did. (School closings, bans on public gatherings, and quarantine were the three chief NPIs Markel tracked.) Neither Copeland nor Harris cites quarantine or isolation as an action taken during the outbreak itself. Harris was explicitly detailing "administrative" public health initiatives to "control" influenza. He was the individual in charge of quarantine. He made no mention of quarantine. Is it plausible, or even conceivable, that he would have made no mention of quarantine if quarantine had actually been imposed?

### Health commissioner's veracity in doubt

Now let's consider Markel's sole source on quarantine: Royal Copeland, who told the *New York Times *that the city was imposing it. The newspaper no doubt quoted Copeland accurately. But who was Copeland? He was a homeopath, not a medical doctor, who said, "Man is a social animal.... Organization is a necessity and my organization is Tammany." Tammany, the most corrupt political machine in American history, had been out of power for several years but regained control of New York City in early 1918. It promptly began putting loyalists into every available patronage job, and it tried to force qualified civil servants out of jobs that were not strictly patronage. For the health department, Tammany initially chose as director someone whom it regarded as a loyalist but not Copeland. But as Tammany moved with increasing aggressiveness to replace highly qualified public health officials with unqualified cronies, after a few months on the job, Tammany's choice for director of the health department quit in protest. When no MD surfaced for the job, Tammany turned to Copeland, not an MD but a true Tammany man.

Why would Copeland lie about the use of quarantine? There were specific reasons in this case, beyond just trying to make himself appear in command. The same day that New York made influenza reportable, Jersey City imposed an actual quarantine. The *Times *reported both actions. It is easy to imagine that this put pressure on Copeland. At any rate, the same day the information about Jersey City was published, he told the *Times *that New York City was using "strict isolation and quarantine," in effect calling Jersey City on quarantine and raising it on "strict isolation."

The larger context makes lying more understandable as well. The United States was at war. Every piece of government-released public information – federal, state, and local – was considered from the perspective of how to keep up morale. The official position of the US government's Committee on Public Information was: "The force of an idea lies in its inspirational value. It matters very little if it is true or false." (Indeed, the pandemic became known as "Spanish influenza" because in all the warring countries, newspapers, which were either censored or intimidated, initially did not report on the disease. Spain was not at war, and its newspapers headlined it. Hence, even though we know the disease appeared in France, Germany, Britain, and the United States before Spain, Spain gave the pandemic its name). Also to maintain morale during the pandemic, national public health officials – people who knew the truth – said, "This is ordinary influenza by another name," and, "You have nothing to fear if proper precautions are taken," and, "This is nothing more than ordinary lagrippe or influenza." Local public health officials all over the country echoed that line.

Copeland and Harris did not, however, lie in the medical journal articles quoted above, which were, as Markel notes, reprints of speeches they made to a meeting of New York City physicians desperate to find ways to alleviate the most lethal disease outbreak in their lives. Copeland would have been scorned out of the room if he had told such lies at such a meeting, to an audience who knew the truth. Therefore they made no mention of quarantine as an action taken during the pandemic.

### Chicago's actions came late

Now to Chicago, which also had a relatively benign experience in the pandemic. According to Markel et al, Chicago tied for the third earliest intervention of the 43 cities he studied, acting on day minus-2, ie, 2 days before the mortality rate exceeded double the baseline rate. We do not know what action Chicago took that day, because the Markel article does not identify any action in any city on any date, nor does it provide any documentation for any of these specific actions. Markel does offer a 1,144-item online bibliography, but the size obscures rather than elucidates, since readers have no way of matching documentation with any particular action.

The article does explain that it uses only three major actions – quarantine, school closings, and general bans on public gatherings – as metrics for NPIs, so Chicago's action should have been one of those three. But according to the Chicago health department's 100-plus-page "Report of an Epidemic of Influenza in Chicago During the Fall of 1918," which is not in Markel's bibliography, only two actions were taken on day minus-2: The state banned public funerals and the city issued orders for teachers to inspect schoolchildren. These actions fall far short of the authors' metrics. The city's most tangible action actually did not occur until day plus-19 (21 days later) which by coincidence was, quoting from the report, "the day when the epidemic was taking its highest death toll," ie, at least several days and possibly a week or more after disease spread had peaked. Only then did Chicago close "theaters of all kinds, cabarets, dance halls, athletic meets, and everything of this kind." But even then, "churches and schools were not closed. Nothing was done to interfere with the morale of the community."

I did not do a systematic review of all 43 cities covered in Markel's article. By pure happenstance, I am familiar enough with events in New York and Chicago to make a judgment on the quality of his assessment of those two only, and I do not know how valid his findings are in the other 41 cities. But his interpretation of data in Chicago and New York does not inspire faith in the rest of his analysis. And since, according to Markel, New York was the earliest city to act and Chicago was tied for third earliest, one wonders whether, even if everything else in the article is correct, errors here would be enough to drop the findings below statistical significance.

Is there another explanation for the relatively benign experiences in New York and Chicago? Possibly. Both cities experienced quite definite spring waves of influenza, which may have immunized some of the population.

Finally, it is important to note that the errors in Markel's article do not disprove the hypothesis that NPIs impacted the course of the pandemic. But any analysis of their historic use must be based on rigorous scholarship. His is not.
